# Effects of slightly acidic electrolyzed water on the microbial quality and shelf life extension of beef during refrigeration

**DOI:** 10.1002/fsn3.779

**Published:** 2018-09-08

**Authors:** Xiaowei Sheng, Dengqun Shu, Xiajun Tang, Yitian Zang

**Affiliations:** ^1^ College of Animal Science and Technology Jiangxi Agricultural University Nanchang China

**Keywords:** beef, microbial quality, shelf life, slightly acidic electrolyzed water

## Abstract

Studies on slightly acidic electrolyzed water (SAEW) for decontamination and shelf life extension of beef are limited. This study aimed to evaluate the effects of SAEW and tea polyphenols (Tpp) on the microbiological, physicochemical, and sensory qualities of fresh beef during storage. The changes in total viable count, thiobarbituric acid content, pH, total volatile basic nitrogen, and sensory scores revealed that the required quality standard of the beef treated with distilled water, Tpp, and SAEW was maintained for up to 6–8, 12–14, and 14–16 days, respectively. These results demonstrated that SAEW could effectively extend the shelf life of beef in comparison with that of other treatments. However, there were no significant differences (*p *>* *0.05) between the untreated and SAEW‐treated group in the content of thiobarbituric acid, suggesting that SAEW does not possess antioxidant activity. Therefore, further studies are required to increase its antioxidant activity. This study suggests that SAEW treatment is an effective and promising method to prolong the shelf life of beef by around 8 days at 4°C.

## 1 INTRODUCTION

Fresh beef is easily contaminated by naturally occurring microorganisms from a variety of sources during processing of all edible carcass tissues (Tango, Mansur, Kim, & Oh, 2014). This might lead to a decrease in the quality and shelf life of beef during storage, and increase health risks. Therefore, it is necessary to develop an effective preservation method that can prolong the shelf life of fresh beef during storage. Storage under refrigerated condition is one of the most commonly employed preservation methods to inhibit the deterioration of fresh meat due to microbial growth, and chemical and biochemical reactions, thus reducing microbial activity and increasing sensory shelf life (Allende, McEvoy, Tao, & Luo, 2009). However, certain complementary refrigeration sanitizing processes prior to the refrigeration storage should be developed and used to improve the safety and quality of fresh vegetables and meat (Li, Ren, Hao, & Liu, 2017).

Recently, various sanitizing processes have been adopted to improve the safety and quality of fresh meat and meat products before refrigeration (Awad, Moharram, Shaltout, Asker, & Youssef, 2012; Guan & Fan, 2010). Among them, wash water and several sanitizing agents, such as chlorine solution, were usually employed for fresh meat refrigeration. But the excessive use of chlorine (Cl_2_) can lead to several environmental problems (Inatsu, Bari, Kawasaki, Isshiki, & Kawamoto, 2005). Furthermore, the consumers are concerned about the use of these chemicals because they might have potentially undesirable effects on human health. Therefore, most studies on the decontamination of fresh meat or vegetables have focused on alternative sanitizing agents to chlorine (Gil, Selma, Lopez‐Galvez, & Allende, 2009; Guentzel, Lam, Callan, Emmons, & Dunham, 2008; Mansur & Oh, 2015).

Slightly acidic electrolyzed water (SAEW) is well recognized as an alternative sanitizer, containing a high concentration of hypochlorous acid, with a pH of 5.0–6.5 (Zang, Li, Bing, & Cao, 2015). It is produced by the electrolysis of dilute hydrochloric acid in a chamber without membrane. When compared to other disinfectants, SAEW has the added advantage of minimized human health and safety issues from Cl_2_ off‐gassing. It is the most environment‐friendly potential alternative to broad‐spectrum microbial decontaminants. Several studies have demonstrated that SAEW could be used as a sanitizer to reduce microbial quality and extend the shelf life of aquatic products and vegetables (Hao et al., 2011; Hricova, Stephan, & Zweifel, 2008; Zang et al., 2017). Li et al. (2017) evaluated the disinfection efficacy of SAEW and strongly acidic electrolyzed water (AEW) on the fresh‐cut lotus roots and demonstrated that SAEW treatment could reduce the natural microbial flora populations significantly. Hao et al. (2011) evaluated the microbial reduction and storage qualities of SAEW on fresh‐cut cilantro, and indicated that SAEW may be a better choice in the storage of freshcut cilantro than AEW. However, a few studies on SAEW for decontamination and shelf life extension of beef are currently being carried out (Tango et al., 2014). Although there have been a study on the application of SAEW, alone or in combination with fumaric acid, in the inactivation of food‐borne pathogens and extending the shelf life of fresh beef. The study mostly focused on the reduction of microbial population and did not discuss the chemical and biochemical properties of beef during storage.

Currently, there are different opinions regarding the antioxidant activity of SAEW. Rahman, Park, Song, Al‐Harbi, and Oh (2012) have reported that SAEW has antioxidant effect and that it can help fresh chicken breast meat to maintain oxidation stability (Thiobarbituric acid, TBA). However, Chen, Xu, Deng, and Huang (2016) have reported that SAEW does not have immediate antioxidant activity and found that the TBA content of the SAEW‐treated samples was not better than unwashed control samples. Therefore, it is necessary to verify if SAEW has antioxidant effect on fresh beef.

This study aimed to evaluate the effectiveness of SAEW on microbiological, physicochemical (TBA, pH, total volatile basic nitrogen), and sensory qualities of fresh beef during storage. Considering that the treatment with tea polyphenols (Tpp) is reported to have bactericidal and antioxidant effects on fresh meat, Tpp were also used for a comparative study.

## 2 MATERIALS AND METHODS

### 2.1 Sample preparation

Fresh boneless beef was purchased from the Wanda food market in Nanchang Province of China and stored in a refrigerator at 4°C prior to its use in the experiments within 3 hr. The meat used in this study was cut into each of 10 ± 0.1 g and 5 × 5 cm^2^ in size using a sterilized sharp knife under a biosafety hood (DH‐920, Beijing East Union Hall Instrument Manufacturing Co., Ltd., Beijing, China) at a room temperature. Samples were used for microbial, pH, TBA, total volatile basic nitrogen (TVB‐N), and sensory analysis.

### 2.2 Sanitizer solution preparation

Slightly acidic electrolyzed water (SAEW) with a pH of 6.29 ± 1.33, ORP of 870‐900 mV, and available chlorine concentration (ACC) of 40 ± 1.27 ppm, used in this study was produced with a nonmembrane generator (Ruiande Biosafety Technology Co., Ltd., Beijing, China) by the electrolysis of NaCl (1 g/L) containing HCl (100 μ/L) solution. The pH and ORP values were measured using a dual scale pH/ORP meter (CON60; Trans‐Wiggens, Singapore). The ACC was determined using a digital chlorine test system (RC‐2Z; Kasahara Chemical Instruments Co., Saitama, Japan). For comparison with SAEW, the Tpp (Jinkelong Biosafety Technology Co., Ltd., Beijing, China) were dissolved in sterile distilled water to obtain a diluted solution of concentration 0.1% and pH 4.83 ± 0.03.

## 3 EXPERIMENTAL PROCEDURE

For washing treatments, the meat samples were dipped in different solutions (SAEW, distilled water, and Tpp) for 5 min at 23°C, respectively, whereas the samples without treatment were used as control. The treated samples were drained, packed in polyethylene bags, and stored at 4°C. The meat samples treated with SAEW and Tpp were then washed for 1 min with 200 ml of neutralizing solution (0.85% NaCl containing 0.5% Na_2_S_2_O_3_) to cease the microbicidal effect of the treatment, and excess sanitizing solutions on the treated meat was removed with sterile paper towel.

Each treatment had three replicates. During the storage, 20 g of sample was collected from each treated sample at an interval of 2 days to evaluate the effect of the preservative, in regard to microbiological, chemical, and sensory analyses. The sample collected immediately after treatment was considered as day 0 sample.

### 3.1 Analysis and determination of quality parameters

#### 3.1.1 Microbiological analysis

Twenty‐five grams of beef was homogenized for 3 min using a homogenizer (Guanshen Biosafety Technology Co., Ltd., Shanghai, China). Following homogenization, the homogenate was mixed with 225 ml of sterile 0.85% sodium chloride solution and agitated for 2 min at low speed. Subsequently, the homogenates were serially diluted and 0.1 ml of each dilution was pipetted onto plate count agar (Aoboxing Bioscience Inc, Beijing, China), which was then incubated at 37°C for 48 hr. The total viable count (TVC) is expressed as log_10_ cfu/g. The untreated beef sample was used as control.

#### 3.1.2 Chemical analyses

The pH of meat was measured using a digital pH meter (CON60; Trans‐Wiggens) after homogenizing 5 g of meat with 10 ml of distilled water.

The content of TVB‐N was estimated by the method of Chen et al. (2016)

The content of TBA was determined by the method of Rahman et al. (2012).

#### 3.1.3 Sensory analysis

The sensory analysis was carried out based on odor, appearance, texture, and overall acceptability by 30 panelists, including staff and students of the Animal Science and Technology Department at the Jiangxi Agricultural University. The panelists were trained prior to the start of the study. The panelists were asked to score independently on a 9‐point hedonic scale by the method of Chen et al. (2016). All samples were evaluated in triplicate, and the samples with scores ≥ 4 were considered acceptable.

### 3.2 Statistical analyses

All experiments were performed in triplicate, and the data are expressed as mean ± standard deviation. The statistical analyses were performed using origin version 9.0. The differences were identified by the analysis of variance (ANOVA) and Duncan's multiple range tests and were considered significant when *p *<* *0.05.

## 4 RESULTS AND DISCUSSION

### 4.1 Changes in microbiological activity

**Figure 1:**
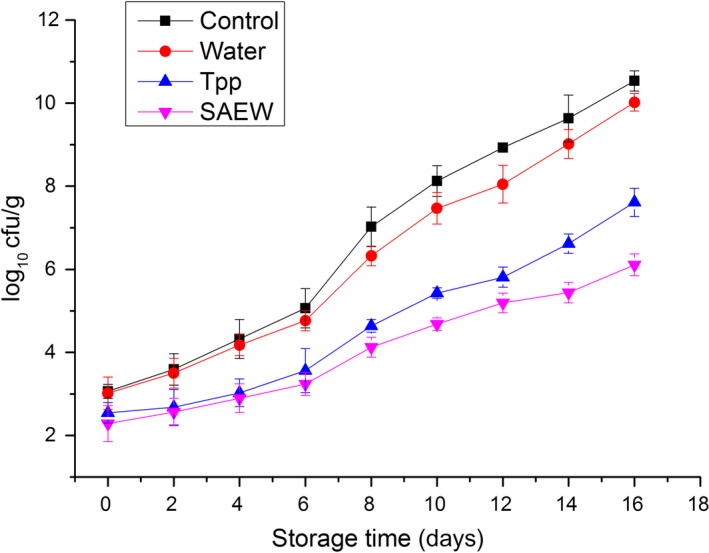
Changes in total viable count of treated and nontreated beef stored at 4°C. Tpp: tea polyphenols, SAEW, slightly electrolyzed water. Vertical bars represent standard error of the mean (*n *≥* *3)

The TVC of fresh beef treated with SAEW and other solutions during storage is presented (Figure 1). The initial TVC of untreated meat was approximately 3.06 log_10 _cfu/g. In comparison with that of the untreated samples, the TVC in the meat treated with distilled water, Tpp, and SAEW decreased to 3.02 ± 0.38, 2.54 ± 0.24, and 2.28 ± 0.43 log_10_ cfu/g, respectively. The SAEW and Tpp treatments exhibited higher disinfectant efficacy compared with that of the distilled water treatment and control (*p *<* *0.05). The TVC increased with storage time in all the samples, but at different rates. As expected, the TVC values of the untreated samples increased at a faster rate than those of all treated samples, indicating the antimicrobial effect of SAEW and Tpp. Similar results have been reported by previous studies (Fabrizio & Cutter, 2004; Mahmoud, Yamazaki, Miyashita, Shin, & Suzuki, 2006). Furthermore, among the treated samples, the sample treated with SAEW exhibited the strongest bactericidal effect on beef. After 3 days, the TVC of distilled water‐treated, Tpp‐treated, SAEW‐treated, and control samples was 4.17 ± 0.25, 3.02 ± 0.33, 2.89 ± 0.35, and 4.32 ± 0.47 log_10_ cfu/g, respectively. The SAEW and Tpp treatments significantly (*p *<* *0.05) slowed down the increase rate of TVC compared with that of the distilled water treatment and control.

In the past few years, the strong disinfectant efficacy of SAEW and its application in disinfection of fruits, vegetables, and meats have been widely studied and reported (Cao, Zhu, Shi, Wang, & Li, 2009; Park, Hung, & Brackett, 2002). The results of this study demonstrate the high disinfectant efficacy of SAEW, which is similar with the findings of previous studies.

The untreated and distilled water‐treated beef samples presented the TVC value of ≥6 log_10_ CFU/g on days 7–16, which is considered as an upper microbiological limit for good quality meat (ICMSF 1986), whereas the TVC value of the SAEW‐ and Tpp‐treated samples was acceptable before days 14 and 12, respectively.

### 4.2 Changes in pH

**Figure 2:**
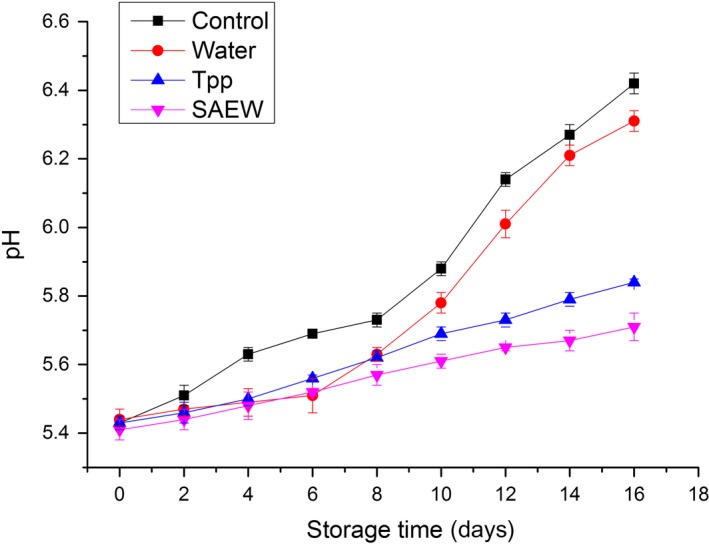
Changes in pH of treated and nontreated beef stored at 4°C. Tpp: tea polyphenols, SAEW, slightly electrolyzed water. Vertical bars represent standard error of the mean (*n *≥* *3)

The mean pH for beef samples ranged from 5.4 to 6.4 during storage at 4°C for control and treated samples (Figure 2). Furthermore, the pH value of meat increased with storage time. This is in agreement with the findings of other studies (Ouattara, Simard, Holley, Piette, & Bégin, 1997). The increase in pH has a relationship with food deterioration on account of the microbial action. The degradation of proteins and production of ammonia can increase pH. Relatively low initial pH values between 5.41 and 5.44 were obtained for all samples, reflecting the good condition of beef. This was consistent with low initial TVC values. The increasing trend of pH of the samples implied the happening of spoilage; however, the pH values of these samples increased at different rates, with the control samples exhibiting the highest rate and SAEW‐treated samples presenting the lowest rate. Consequently, the pH increased to 6.42 ± 0.03, 6.31 ± 0.03, 5.84 ± 0.01, and 5.71 ± 0.04 for the control, distilled water‐treated, Tpp‐treated, and SAEW‐treated samples on day 16, respectively. The results suggest that SAEW has inhibitory effects on spoilage microorganisms, slowing down the increase in pH and delaying the generation of basic nitrogen compounds, which are better than those of Tpp.

### 4.3 Changes in TVB‐N

**Figure 3:**
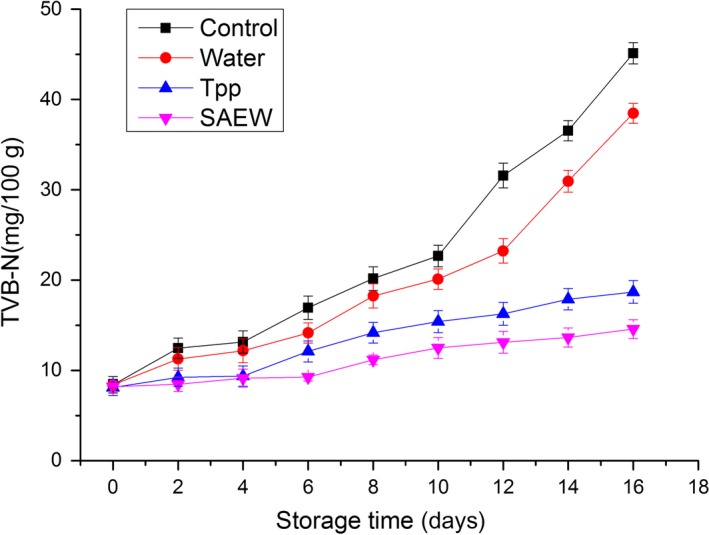
Changes in total volatile basic nitrogen (TVB‐N) of treated and nontreated beef stored at 4°C. Tpp: tea polyphenols, SAEW, slightly electrolyzed water. Vertical bars represent standard error of the mean (*n *≥* *3)

The TVB‐N is mainly composed of ammonia and primary, secondary, and tertiary amines (Gill, 1983). It results from the degradation of proteins and nonprotein nitrogenous compounds, produced chiefly due to the microbial activity. It is regarded as an important and sensitive indicator of freshness of meat during storage (Veberg et al., 2006). The TVB‐N values of the samples during storage are shown in Figure 3. The initial TVB‐N values were 8.40 ± 0.91, 8.40 ± 0.41, 8.12 ± 0.89, and 8.19 ± 0.63 mg/100 g for control, distilled water‐treated, Tpp‐treated, and SAEW‐treated samples, respectively. The TVB‐N steadily increased with storage time in all the treatment groups. However, the increase in TVB‐N was substantially (*p *<* *0.05) slower in the Tpp‐ and SAEW‐treated samples than in the distilled water‐treated and control samples, with the SAEW and Tpp treatments presenting the slowest increasing rate. This was consistent with the TVC and pH values. The concentration of TVB‐N of the control group rapidly increased to 16.94 ± 1.29 mg/100 g on day 6 of storage, whereas lower values of 14.14 ± 1.11, 12.10 ± 1.17, and 9.25 ± 0.43 mg/100 g were observed in distilled water, Tpp, and SAEW treatment groups.

Studies have reported that the maximum allowable upper TVB‐N limit for beef is 20 mg/100 g. Based on this acceptability limit, the treatment with Tpp and SAEW significantly extended the shelf life of beef from 8 (control group) to 16 days. The treatment with Tpp and SAEW suppressed the formation of TVB‐N most effectively.

### 4.4 Changes in TBA content

**Figure 4:**
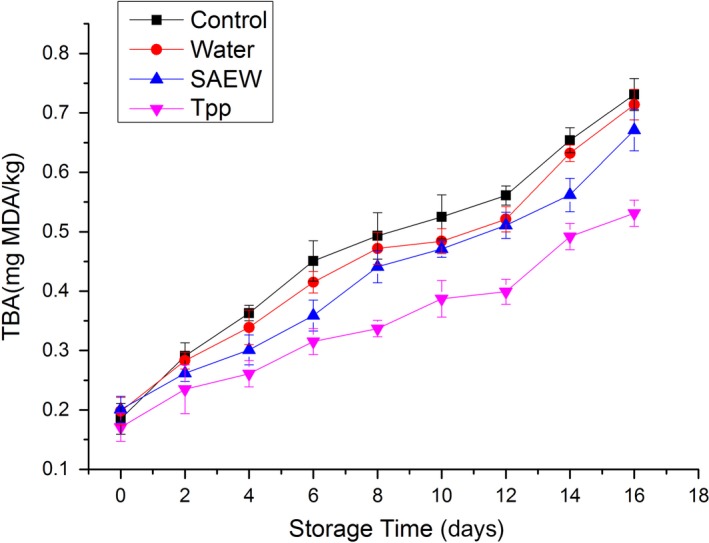
Changes in thiobarbituric acid (TBA) of treated and nontreated beef stored at 4°C. Tpp: tea polyphenols, SAEW, slightly electrolyzed water. Vertical bars represent standard error of the mean (*n *≥* *3)

The content of TBA represents the degree of lipid oxidation of food (Campo et al., 2006). Lipid oxidation is an important factor of oxidative deterioration of meat, leading to the formation of off‐flavor and off‐odor, thus limiting the shelf life (Patsias, Chouliara, Badeka, Savvaidis, & Kontominas, 2006). The changes in the content of TBA of treated and untreated beef during storage are depicted in Figure 4. The content of TBA of all the tested samples was similar at the beginning of storage. The results showed that there were no significant differences (*p *>* *0.05) in the initial TBA content between the untreated and treated samples, with an initial TBA content between 0.17 and 0.18 mg MDA/kg. An increasing trend in TBA content was observed with increase in storage time for all the samples, although at different rates. The results showed that the TBA values of the four groups increased gradually from the initial to 0.73 ± 0.03, 0.71 ± 0.02, 0.67 ± 0.03, and 0.53 ± 0.02 mg MDA/kg during storage in the control, distilled water‐treated, Tpp‐treated, and SAEW‐treated samples, respectively.

There were significant differences (*p *<* *0.05) between the untreated and Tpp‐treated samples, which is consistent with the findings of a previous study (Lu et al., 2010). The Tpp play an important role in protein precipitation and enzyme inhibition and have beneficial antibacterial and anti‐oxidative activities (Khan & Mukhtar, 2007). They are used as preservatives and antioxidants in food industry, especially to preserve meat. The antioxidant effect of Tpp is mainly due to the inhibition of enzyme activities and free radical scavenging ability and therefore preventing lipid oxidation (Frei & Higdon, 2003).

We observed that there were no significant differences (*p *>* *0.05) between the untreated and SAEW‐treated samples, suggesting that SAEW has no antioxidant activity. This result is consistent with that of Chen et al. (2016) who reported that SAEW has no immediate antioxidant activity. However, this is different from the findings of Rahman et al. (2012), who reported that SAEW, which contains ^−^OH and HOCl, has antioxidant effect, and can maintain the oxidation stability of poultry meat. This difference in results might be because the poultry meat is particularly prone to oxidation than beef, as it contains relatively high levels of unsaturated fatty acids and low levels of natural antioxidants. Xuan et al. (2017) have reported that SAEW in the form of ice can maintain relatively low TBA contents during the preservation of squid. This indicates that SAEW ice might be a new approach to ensure the antioxidant activity and control the deterioration of quality of beef during storage. Further studies are required to increase the antioxidant activity of SAEW on beef.

### 4.5 Changes in sensory properties

**Figure 5:**
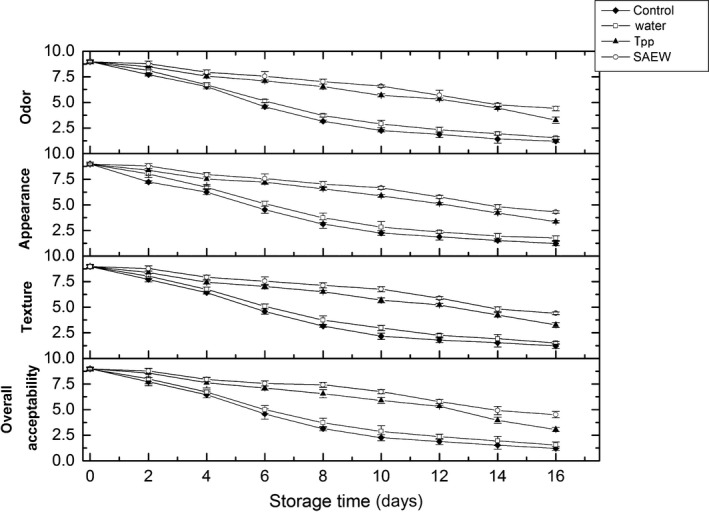
Changes in sensory properties of treated and nontreated beef stored at 4°C. Tpp: tea polyphenols, SAEW, slightly electrolyzed water. Vertical bars represent standard error of the mean (*n *≥* *3)

The sensory evaluation of odor, appearance, texture, and overall acceptability was performed by the panelists, and the average scores are illustrated in Figure 5. During storage, there was a decrease in the sensory properties of all the treated samples, displaying a progressive loss of freshness of meat. The results also revealed that the SAEW and Tpp treatments can maintain the sensory properties of meat better than the control and distilled water treatments. Furthermore, there were significant differences (*p *<* *0.05) between the SAEW‐ and Tpp‐treated samples on day 16 of storage. At the end of storage (day 16), the odor, appearance, texture, and overall acceptability scores of the SAEW‐treated samples were approximately 4.52 ± 0.12, 4.43 ± 0.13, 4.31 ± 0.13, 4.42 ± 0.25, respectively, which were higher than those of other treatments, including the distilled water, Tpp, and control. The changes in the overall quality of beef showed that the SAEW treatment had the advantage of maintaining the overall quality of beef.

## 5 CONCLUSION

Overall, the microbial, chemical, and sensory properties correlated highly with the freshness of beef. The results of the present study suggest that SAEW is a potential method to extend the shelf life of fresh meat.
